# Spatial variability in size and lipid content of the marine copepod *Calanus finmarchicus* across the Northwest Atlantic continental shelves: implications for North Atlantic right whale prey quality

**DOI:** 10.1093/plankt/fbad047

**Published:** 2023-12-09

**Authors:** Laura K Helenius, Erica J H Head, Phoebe Jekielek, Christopher D Orphanides, Pierre Pepin, Geneviève Perrin, Stéphane Plourde, Marc Ringuette, Jeffrey A Runge, Harvey J Walsh, Catherine L Johnson

**Affiliations:** Fisheries and Oceans Canada, Bedford Institute of Oceanography, P.O. Box 1006, Dartmouth, NS B2Y 4A2, Canada; Fisheries and Oceans Canada, Bedford Institute of Oceanography, P.O. Box 1006, Dartmouth, NS B2Y 4A2, Canada; School of Marine Sciences, University of Maine, 5706 Aubert Hall, Orono, ME 04469-5706, USA; NOAA, NMFS, Northeast Fisheries Science Center, 28 Tarzwell Drive, Narragansett, RI 02882, USA; Fisheries and Oceans Canada, Northwest Atlantic Fisheries Centre, P.O. Box 5667, St. John's, NL A1C 5X1, Canada; Fisheries and Oceans Canada, Institut Maurice-Lamontagne, P.O. Box 1000, Mont-Joli, QC G5H 3Z4, Canada; Fisheries and Oceans Canada, Institut Maurice-Lamontagne, P.O. Box 1000, Mont-Joli, QC G5H 3Z4, Canada; Fisheries and Oceans Canada, Bedford Institute of Oceanography, P.O. Box 1006, Dartmouth, NS B2Y 4A2, Canada; School of Marine Sciences, University of Maine, 5706 Aubert Hall, Orono, ME 04469-5706, USA; NOAA, NMFS, Northeast Fisheries Science Center, 28 Tarzwell Drive, Narragansett, RI 02882, USA; Fisheries and Oceans Canada, Bedford Institute of Oceanography, P.O. Box 1006, Dartmouth, NS B2Y 4A2, Canada

**Keywords:** calanoid, energy content, oil sac

## Abstract

Copepod size and energy content are influenced by regional and seasonal variation in temperature and food conditions, with implications for planktivorous consumers such as the endangered North Atlantic right whale (*Eubalaena glacialis*). Historical data (1990–2020) on *Calanus finmarchicus* stage CV copepodite prosome length and oil sac metrics were analyzed to determine the extent of variation in individual body size and estimated lipid and energy content in five regions of the Northwest Atlantic continental shelves [Gulf of Maine (GoM), Scotian Shelf (SS), Gulf of St. Lawrence (GSL), St. Lawrence Estuary (SLE) and Newfoundland Shelf]. Large-scale spatial patterns in size and lipid content were related to latitude, indicating that *C. finmarchicus* CV in the GSL and SLE were historically larger in body size, and had significantly higher lipid content compared with those in the GoM and the SS. The observed patterns of *C. finmarchicus* CV size and lipid storage capacity suggest that regional variation in whale prey energy content can play a role in the suitability of current and future whale foraging habitats in the Northwest Atlantic, with the larger lipid-rich individuals in the GSL providing a high-quality diet compared with those in southern areas.

## INTRODUCTION

The nutritional value of prey is determined by both its quantity and quality. Prey abundance and biomass define prey quantity, whereas prey quality can be assessed from proximate composition, essential nutrient content and energy content of prey (Elser *et al*., 2000; Hildebrand *et al.*, 2022). In temperate, sub-arctic and arctic marine ecosystems, a significant determinant of prey item energy richness is lipid content, and lipids synthesized by and stored in prey fuel major pathways of marine food webs (Benson and Lee, 1972; Hirche, 1996; Kattner and Hagen, 2009). In these latitudes, copepods of the genus *Calanus* accumulate and store large reserves of energy-rich wax esters in a membrane-bound oil sac extending through the length of the body (Marshall and Orr, 1972; Lee, 1975; Kattner and Krause, 1987; Lee *et al.*, 2006). In *Calanus finmarchicus*, one of the most ubiquitous species of copepods in the North Atlantic (Head *et al.*, 2013; Melle *et al.*, 2014, 2015), the oil sac is at its largest in stage CV copepodites, which typically require large lipid stores to diapause at depth. This diapausing trait plays an essential role in the availability of marine lipids by concentrating energy-rich lipids from short phytoplankton blooms into a biological “battery”, which provides higher trophic levels with a source of energy over a longer period (Record *et al.*, 2018). The combination of the energetic value and the buoyancy characteristics of stored lipids is thought to be essential in the success of this diapausing stage (Irigoien, 2004).

Large spatio-temporal variability exists in the abundance and distribution of *C. finmarchicus* populations in the western North Atlantic, linked to various environmental and physical influences (e.g. Melle *et al.*, 2014; Pepin *et al.*, 2015; Sorochan *et al.*, 2019). Much of the variation in copepod biomass in the western North Atlantic is derived from variation in the abundance of large, dominant species, yet temperature-driven changes in individual body size may also contribute (Sorochan *et al.*, 2019). Large-scale patterns of *C. finmarchicus* body size can be represented by a negative linear relationship between copepod prosome length (*PL*) and temperature (Wilson *et al.*, 2015). Therefore, increasing ocean temperatures associated with climate change could be expected to reduce the size of individuals (Campbell *et al.*, 2001a; Forster and Hirst, 2012; Wilson *et al.*, 2016) and presumably their energy content, potentially reducing their quality as prey. Hence there is a need to quantify variation in individual lipid content of copepods at larger regional scales to be able to assess changes in subsequent energy availability for zooplanktivorous consumers. One consumer of large energy-rich copepods is the endangered North Atlantic right whale (NARW, *Eubalaena glacialis*), which feeds primarily on *Calanus* spp. (Stone *et al.*, 1988; Kann and Wishner, 1995; Woodley and Gaskin, 1996). In their traditional foraging habitats where other *Calanus* spp. are absent or rare, NARW rely largely on late-copepodite or adult stages of *C. finmarchicus*, and are particularly well adapted to capturing these prey (Mayo *et al.*, 2001; Baumgartner and Mate, 2003), as well as efficiently metabolizing their wax ester-rich lipids (Wishner *et al.*, 1995; Swaim *et al.*, 2009). NARW reside off the east coast of North America, from Florida to Newfoundland (Winn *et al.*, 1986; Kraus and Rolland, 2007). In their northern foraging grounds (i.e. north of the Mid-Atlantic Bight), movements of NARW appear to track the abundance of *Calanus* spp., with several studies associating variations in abundance and availability of *Calanus* spp. with NARW distribution (Pendleton *et al.*, 2009; Davies *et al.*, 2015; Meyer-Gutbrod and Greene, 2018; Ganley *et al.*, 2019; Plourde *et al.*, 2019; Record *et al.*, 2019; Meyer-Gutbrod *et al.*, 2022) or calving rate (Greene and Pershing, 2004; Meyer-Gutbrod *et al.*, 2015). Historically, NARW have moved from foraging grounds in the western Gulf of Maine (GoM) in winter and spring to feed in the eastern GoM and Scotian Shelf (SS) in the summer and autumn (Baumgartner and Mate, 2005), leading to the designation of a region in the GoM (Northeastern US Foraging Area) as well as Grand Manan Basin in the Bay of Fundy and Roseway Basin on the SS as critical NARW habitats in the Northwest Atlantic (Kraus and Rolland, 2007; Brown *et al.*, 2009; NMFS, 2015). However, the migration patterns of NARW have changed since 2008–2010 (Record *et al.*, 2019; Quintana-Rizzo *et al.*, 2021; Durette-Morin *et al.*, 2022; Meyer-Gutbrod *et al.*, 2022). NARW have been documented year-round in southern New England waters on the southern edge of the GoM (Davis *et al.*, 2017), and almost 50% of the reproductive female right whale population has been sighted foraging in the winter (Quintana-Rizzo *et al.*, 2021). Summer and autumn foraging distributions have shifted, with more individuals observed in the Gulf of St. Lawrence (GSL) (Stokstad, 2017; Simard *et al.*, 2019), particularly the southern GSL. Hypotheses suggest the changes in ocean circulation and a warming-driven decline of *C. finmarchicus* at the southern end of its distribution range in the NW Atlantic drove whales to move northward into regions where their presence was not anticipated, resulting in increased NARW mortality from vessel strikes and fishing gear entanglements prior to measures implemented in 2018 to minimize these interactions (Daoust *et al.*, 2017; Stokstad, 2017; Meyer-Gutbrod and Greene, 2018; Pettis *et al.*, 2018). The timing of declines in biomass of *Calanus* spp. in GoM and on SS is consistent with the rationale that a reduction in prey abundance has contributed to changes in the spatial distribution of NARW (Sorochan *et al.*, 2019). Regardless, each of the current and past feeding areas (GoM, SS and GSL) includes deep basins or slope water areas identified as *C. finmarchicus* population centers, where diapausing populations normally exceed 1.5 × 10^4^ individuals ∙ m^−2^ (Melle *et al.*, 2014): this is also the case for the waters of the Newfoundland Shelf (NFL), a potentially overlooked foraging habitat.

Conservation efforts for NARW require a better understanding of energetics provided by foraging areas, and model input data to enable prediction of the energetics of current and future whale foraging habitats. Despite potentially significant effects of energetic variations in *C. finmarchicus* as NARW prey, there are relatively few studies on spatial differences in individual size and lipid metrics in NARW feeding areas. Michaud and Taggart (2007, 2011) reported significant changes in *C. finmarchicus* energy content on both spatial and temporal scales in the lower Bay of Fundy around the critical NARW foraging habitat of Grand Manan Basin (Brown *et al.*, 2009), which were also correlated with NARW presence. In the same habitat, McKinstry *et al.* (2013) found the substantial variation in *C. finmarchicus* lipid and energy content to be reflected in those of zooplanktivorous herring. Individual energetic variation, largely defined by lipid content in lipid-rich copepods such as *C. finmarchicus*, can therefore be an important factor to take into consideration when using bioenergetic models to assess potential foraging habitats for zooplanktivores and evaluation of trophic transfer.

Species of *Calanus* exhibit notable plasticity in body size and energy content in response to their fluctuating environments (Falk-Petersen *et al.*, 2009), so their relative energetic values at different latitudes are expected to show considerable variability. Both spatial and temporal variations in lipid content have been found in several intraregional studies (Michaud and Taggart, 2007, 2011; Pepin and Head, 2009; McKinstry *et al.*, 2013). It is therefore likely that there are high levels of inter-regional spatial variability in individual copepod size and lipid content metrics that determine prey quality in different NARW feeding areas, which may contribute to changes in the suitability of these regions for sustaining populations of NARW*.* The overarching aim of this study was to quantify the degree of inter-regional variation in the individual size and lipid content of *C. finmarchicus*, specifically those of the main diapausing copepodite V (CV) stage, in current and potential future NARW feeding grounds in the western North Atlantic. To do this, we analyzed a subset of a large historical dataset compiled from the Northwest Atlantic over the last three decades (1990–2020, Helenius *et al.*, 2022) to determine regional and temporal differences in individual *C. finmarchicus* CV lipid content. Specifically, we aimed to answer the following questions: (i) Have there been season-specific spatial differences in mean *PL* of *C. finmarchicus* CV in current (GoM, GSL, SS) and potential (NFL) foraging regions over the last three decades? (ii) What is the overall maximum capacity for lipid storage, measured as oil sac volume (*OSV*), in these CV and (iii) Are there variations in the realized CV individual lipid storage and inferred energy content among the different regions over the last three decades? Our results can be used as inputs for spatial bioenergetic models, as well as to further shed light on recent developments in NARW distribution and movements.

## METHOD

**Fig. 1:**
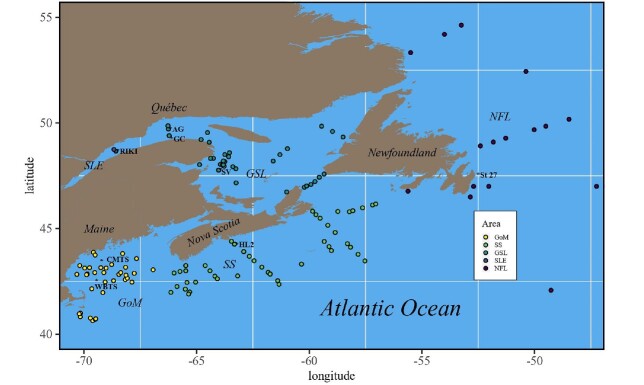
Map of the study area with the main regions of comparison in the Northwest Atlantic Ocean (GoM, GSL, SLE, SS and the NFL region). Asterisks indicate approximate positions of the fixed stations in each region. Easternmost sampling points in NFL area are not shown (47°N, 43°W and 47°N, 43°4’W).

Our methodological goal was to compare the available historic data from Northwest Atlantic regions in terms of a readily available body size metric (*PL*), and to relate this metric to lipid content (oil sac lateral length, area and volume—*OSL, OSA* and *OSV,* respectively). The main regions where data have been collected included the GoM, SS, GSL and its lower estuary [St. Lawrence Estuary (SLE)] and NFL (Fig. 1). The data were compiled from a diverse range of historical datasets, which included *C. finmarchicus* CV body size and/or lipid content data spanning the years 1990–2020 (Helenius *et al.*, 2022). Observations originate from a variety of monitoring programs, including the Fisheries and Oceans Canada Atlantic Zone Monitoring Program (AZMP) in the GSL (sampling lines throughout the Gulf, as well as the Rimouski sampling station in the SLE), SS and NFL; GoM stations operated by the Northeastern Regional Association of Coastal Ocean Observing systems and the US National Oceanic and Atmospheric Administration (NOAA) Marine Resources Monitoring, Assessment and Prediction and Ecosystem Monitoring (MARMAP and EcoMon) surveys in the GoM, including Nantucket Shoals. Samples were collected from time series stations in the regions including Halifax-2 (HL2) in SS, Rimouski (RIKI) in SLE, and Anticosti Gyre, Gaspé Current and Shediac Valley in GSL (Fig. 1), and at stations on seasonally sampled transects (AZMP) or in shelf survey strata (MARMAP, EcoMon). Sampling stations in the Cabot Strait transition zone between GSL and SS were included in GSL data. GoM data included samples collected at a time series station in Wilkinson Basin (WBTS), and at the Coastal Maine Time Series station located at the landward margin of the Maine Coastal Current, as well as from stations along transects and at opportunistic stations in the coastal and Wilkinson Basin areas (Runge and Jones, 2012; Runge *et al.*, 2015; Fig. 1). NFL data were from the time series station Station-27 (ST 27, Fig. 1), and stations along shelf transects (AZMP; Pepin and Head, 2009).

Preliminary data exploration of the historical dataset resulted in the decision to exclude some subsets of data because of large numbers of anomalously low *PL* values (e.g. all of the values in the subset were below the overall regional mean, and > 50% were more than 2 SD below the overall regional mean), which indicated potential problems with data quality. All remaining observations with an estimate of either size metric (*PL, OSL, OSA* or *OSV*) were included in the analyses. Data that included *PL* measurements spanned the years 1994–2020 (*n* = 26 029), with an uneven distribution of *PL* observations among decades (1990–1999, 2000–2009, 2010–2020) and regions. Observations from the GoM were most abundant (30% of all *PL* observations), while data from the SLE and NFL each represented 13% of all observations. Approximately 51% of the *PL* observations were from 2000–2009, and data that included *OSV* measurements spanned the years 2000–2019 (*n* = 15 047). Approximately 94% of the *OSV* observations were from individuals with a *PL* between 2 and 3 mm. Data that additionally included *DW* measurements were from years 2006–2019 (*n* = 1346), and included all regions except NFL. Misidentified *Calanus glacialis* could result in a bias in the estimated mean *PL* and *OSV*, particularly in the SS, GSL, SLE and NFL regions, but focusing our key analyses on the 2–3 mm size class of individuals should minimize their influence, since *PL* of 2.85–2.9 mm is often used as a cutoff for identification of *C. finmarchicus* (Plourde *et al.*, 2001; Parent *et al.*, 2011). Furthermore, *C. finmarchicus* is generally substantially more abundant than *C. glacialis* in the SS, GSL and NFL (Head *et al.*, 1999; Plourde *et al.*, 2001; Parent *et al.*, 2011; Pepin *et al.*, 2011; Casault *et al.*, 2020a).

Sampling gear and methodology were relatively consistent in all areas, with net diameters and mesh sizes ranging from 0.6–1 m and 200–333 μm, respectively. In most cases, zooplankton were sampled using 0.75 m diameter ring nets equipped with 200 or 333 μm mesh and a suspended flowmeter, towed vertically from near-bottom (within ~5 m of station bottom depth) or from 1000 m (for bottom depths >1000 m) to the surface at a rate of ~1 m s^−1^ (i.e. AZMP protocol; Mitchell *et al.*, 2002). Some samples were preserved in a 4% seawater-buffered formaldehyde, while approximately half of the dataset originated from imaging live or live-frozen samples. Records of both preserved and live samples were available from each region, but all oil sac metrics from SS and NFL originated from live samples. In the GoM, opportunistic sampling of live animals was conducted using a 1 m diameter ring net equipped with 333-μm mesh, towed vertically from within ~5 m of sampling point bottom depth at a speed of 0.25 m s^−1^ to a maximum depth of 100 m. Maximum sampling depths for the full dataset ranged from 25 to 1000 m.

Image analysis was used to determine size and lipid content metrics. Because of the long-term nature of the various monitoring projects involved, photo imaging was performed by several analysts using various types of microscopes and image analysis systems. In general, the ideal photograph for digital analysis of lipid content is a clear lateral view and outline of the prosome and oil sac, as well as a visible urosome to enable staging of the copepod (Miller *et al.*, 1998). The procedure for acquiring measurements of *PL* and *OSA* utilizes the simple line tool available in each imaging system. *PL* is defined as the linear distance from the tip of the cephalothorax to the tip of the last thoracic segment in lateral view. *OSA* was estimated by manually outlining the perimeter of the oil sac using the free hand selections tool of each imaging system, counting the number of pixels within the perimeter outline, and then converting pixel counts to mm^2^ using a calibration factor (Miller *et al.*, 1998; Vogedes *et al.*, 2010). *OSV* was estimated using *OSA* and *OSL* following Miller *et al.* (1998):


(1) \begin{equation*} OSV=\frac{\pi{OSA}^2}{4 OSL} \end{equation*}

To support seasonal comparability among individuals in different regions, the samples were coarsely segregated into predetermined phases by sampling month based on proportions of life history stages in the overall *C. finmarchicus* population. Phases included (i) emergence from diapause and molting to CVI adults (months where adults were >10% of the total abundance of copepodite stages), termed “activation,” (ii) growth and development (increased proportion of CV in the copepodite population and interim between emergence and diapause onset), termed “growth,” (iii) diapause onset (proportion of CVs in the population is half its annual maximum), termed “onset” and (iv) diapause (annual maximum proportions of CV in the population), termed “diapause.” These phases were defined so that expected lipid content was either minimal (activation), accumulating (growth), or maximal (onset, diapause) (Hirche, 1996). They were determined separately for each region (GSL, SLE, GoM, SS, NFL) from *C. finmarchicus* life history data and corroborated by comparing with the region-specific estimated timing of the spring phytoplankton bloom (Zakardjian *et al.*, 2003 and references within; Johnson *et al.*, 2008; Ji, 2011; Casault *et al.*, 2020b; Blais *et al.*, 2021) (Table I). Approximately 60% of all *PL* observations in our dataset were from individuals in onset or diapause phases, with an overall mean (SD) *PL* of 2.38(0.24) mm (*n* = 11 152).

**Table I:** Life phase division

*Phase*	***GoM***	***SS***	***GSL***	***SLE***	***NFL***
*a) CV emergence from diapause/maturation into CVI (“activation”)*	Dec–Feb	Dec–Feb	Jan–**Apr**	Apr–Jun	Dec–Apr
*b) CV growth and development during spring bloom (“growth”)*	Mar–**May**	Mar–**May**	**May**–**Jul**	Jul–Aug	May–Jul
*c) CV diapause onset (“onset”)*	**Jun**–**Aug**	**Jun**–**Aug**	**Aug**–Oct	Sept	Aug–Nov
*d) CV diapause (“diapause”)*	**Sept**–Nov	**Sept**–Nov	Nov–Dec	Oct–Mar	(Aug–Nov)

Region-specific division of months based on life phases of varying lipid content (a–d) in *C. finmarchicus* stage CV copepodites in the GoM, the subregions of the GSL [gulf (GSL) and SLE], SS and NFL. Months in bold indicate time periods that are significant in terms of NARW sightings in each region.

To determine temporal and regional copepod size differences, individual *PL* during the phases of maximum lipid content (onset and diapause) were compared within and among the different regions by decade (1990–1999, 2000–2009, 2010–2020) using linear models of the following form: 


(2) \begin{equation*} PL = \alpha + \beta + \varepsilon \end{equation*}

where α is the intercept, ε is the error and β is the categorical predictor of either region or decade to compare *PL* among or within regions, respectively. Approximate normal distributions and homoscedasticity of residuals were confirmed through diagnostic plots (Q-Q and scale-location, respectively). When significant terms were found, spatial and temporal differences were examined with *post hoc* tests (Tukey procedure).

Oil sac metrics in copepods can range from zero to an upper limit determined by each individual’s body volume, which increases with PL (e.g. Miller *et al.*, 2000 ; Pepin and Head, 2009). Although *OSA* can be used as a proxy for lipid content using conversions, such as calculated by Vogedes *et al.* (2010), *OSV* is more appropriate for inferring energy content from irregularly shaped oil sacs (Davies *et al.*, 2012). Therefore, we chose to use *OSV* as the lipid metric in our analyses. The maximum potential *OSV* at any given *PL* (termed *OSV*_max_) was estimated as the 0.95 quantile of *OSV* using quantile regression for the combined data available in lipid-rich phases (i.e. onset and diapause) and from all regions (years 1999–2019). To examine potential region-specific differences in proportions of the CV population accumulating the maximum potential amount of lipid at a given size, we used the quantile regression model to calculate predicted *OSV*_max_ for each datapoint and the deviance of actual observed values of *OSV* from *OSV*_max_ as a percentage (hereafter referred to as “oil sac fullness”). This oil sac fullness was used to compare formalin-preserved and live individuals in sampled populations in different regions using linear models as above (equation 2). Extreme outliers (observations where oil sac fullness >150% were 3 SD outside of the overall mean and represented <0.05% of the observations) were removed from further analyses. Empirical cumulative distribution function plots were also used to visualize the regional differences in proportions of copepods reaching ≤50% (“low lipid”) and ≥100% (“high lipid”) of *OSV*_max_.

To obtain ecologically meaningful average energy content estimates from lipid data, we modeled individual copepod *OSV* as a function of *PL* in the two diapause phases using generalized linear models (GLM) with a gamma-distributed response variable (*OSV*) and a log-link function of the following form: 


(3) \begin{equation*} OSV = \exp (\beta_{O} + \beta_{PL}+ \varepsilon) \end{equation*}

Because the historical dataset contained data from both live and formalin-preserved samples, a model including “region” and “preservation state” (live or formalin-preserved) as predictor variables was constructed (equation 4) prior to testing region-specific models. 


(4) \begin{equation*} OSV = \exp (\beta_{0} + \beta_{PL} + \beta_{REGION} + \beta_{PRESERVATION} + \varepsilon) \end{equation*}

Preliminary analyses demonstrated significant effects of region and preservation state on the estimated *OSV*, so separate estimates were made to avoid bias and potential errors. Residual and null deviance of each model was noted to assess the general goodness of fit. Because of consistent model misfits in the lower and upper bounds of our data range, the GLMs for some regions appeared to overestimate *OSV* in the smallest and largest size classes of copepods (Figs S1, S2). To more adequately model the *PL-OSV* relationship outside of the main 2–3 mm size class of copepods and to minimize uncertainty in estimating the central tendency from the GLMs, we additionally used quantile regression to model the relationships in terms of 0.1, 0.5 and 0.9 quantiles (Figs S1, S2, S3). Estimates from the 0.5 quantile regressions were presented in addition to those from the GLMs.

Using the models for each region, a predicted *OSV* was calculated for the mean *PL* for each decade where data were available. An estimate of mass total lipid (*TL*) in the oil sac for each predicted *OSV* was derived for comparison with previously published values using a lipid density of 0.9 g mL^−1^ (Miller *et al.*, 1998; Visser and Jónasdóttir, 1999). Individual energy content from lipid (*EC*_ind_) for each average-sized individual was estimated assuming lipid energy content of 39.5 kJ g^−1^ (Comita *et al.*, 1966; Lamprecht, 1999; Davies *et al.*, 2012). The *EC*_ind_ value was presumed to be an accurate representation of total energy content in individuals with high lipid content in the onset and diapause phases (Davies *et al.*, 2012), but did not account for energy derived from non-lipid body mass. It should therefore be interpreted with caution in CV with lower size-adjusted oil sac fullness. A limited subset of data from the second and third decades of the dataset (2006–2019) was additionally analyzed to estimate dry weight-specific energy content from lipid (J mg^−1^  *DW*, *EC_DW_*) using copepod *OSV* and individual *DW* observations, and the *TL* and *EC*_ind_ conversions above. The resulting regional values were compared using linear models as above.

## RESULTS

**Fig. 2:**
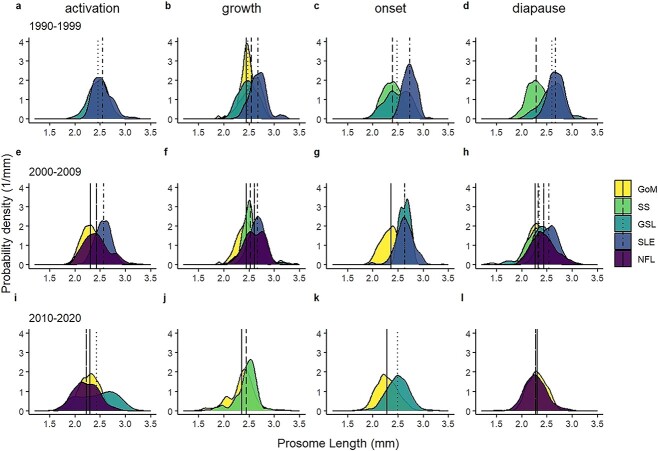
Smoothed (kernel density estimate) distribution of *PL* of *C. finmarchicus* stage CV in the four predetermined phases (activation, growth, onset and diapause) over three decades (a-d: 1990-1999; e-h: 2000-2009; i-l: 2010-2020) in regions of the Northwest Atlantic (GoM, SS, GSL, SLE and NFL). Vertical lines show regional means for each region. Note that in (g), GSL and SLE means overlap directly to produce dot-dash line at *PL* of 2.63 mm.

Linear models comparing *PL* within region and phase (onset and diapause) did not indicate consistent temporal patterns in body size changes (Table II). In the GoM, CV were larger in 2000–2009 compared with 2010–2020 in the onset phase, but smaller in the diapause phase (Table II). On the SS, they were smaller in the diapause phase in 1990–1999 compared with 2000–2009 (Table II). Conversely, in GSL and SLE, in both the onset and diapause phases CVs were smaller in more recent decades relative to 1990–1999, where data were available for comparison, with the exception of GSL onset phase, where *PL* was higher in 2000–2009 compared with the other time periods (Table II, Fig. 2).

**Table II:** Regional comparison of copepodite body size

Region	Mean *PL* (SD)													
	**1990–1999** (*1*)				**2000–2009** (*2*)				**2010–2020** (*3*)				*F_df (Decade)_*	
	*Onset*	*N*	*Diapause*	*N*	*Onset*	*N*	*Diapause*	*N*	*Onset*	*N*	*Diapause*	*N*	*Onset*	*Diapause*
GoM (*A*)					2.36 (0.21) *C, D,* 3^*^^*^^*^	978	2.27 (0.21) B, C, D, E, 3^*^	485	2.28 (0.21) *C,* 2^*^^*^^*^	1 451	2.31 (0.19) *D,* 2^*^	1 186	*F*_1, 2427_ = 84.04	*F*_1, 1520_ = 11.38
SS (*B*)	2.38 (0.19) *C, D*	948	2.28 (0.18) *C, D,* 2^*^^*^	607			2.33 (0.22) *A, D, E,* 1^*^^*^	630						*F*_1, 1235_ = 14.88
GSL (*C*)	2.48 (0.23) *D, B,* 2^*^^*^	238	2.60 (0.22) *B,* 2^*^^*^^*^	25	2.63 (0.1) *A,* 1^*^, 3^*^	18	2.35 (0.24) A, D, E, 1^*^^*^^*^	4 470	2.49 (0.22) *A,* 2^*^	237			*F*_2, 488_ = 3.88, *P* < 0.05	*F*_1, 4493_ = 26.18
SLE (*D*)	2.73 (0.13) *B, C,* 2^*^^*^^*^	134	2.67 (0.14) *B,* 2^*^^*^^*^	144	2.63 (0.19) *A,* 1^*^^*^^*^	491	2.54 (0.2) A, B, C, E, 1^*^^*^^*^	1 213					*F*_1, 382_ = 26.91	*F*_1, 1127_ = 50.36
NFL (*E*)							2.44 (0.25) A, B, C, D, 3^*^^*^^*^	1 676			2.28 (0.22) *A,* 2^*^^*^^*^	493		*F*_1, 2167_ = 169.7
*F_df (Region)_*	*F*_2, 1305_ = 179.7		*F*_2, 749_ = 258		*F*_2, 1255_ = 196.4		*F*_4, 8265_ = 192.9		*F*_1, 1684_ = 189.6		*F*_1, 1 528_ = 7.763, *P* < 0.01			

Regional mean (with standard deviation) PL (mm) per decade and phase (onset or diapause) of *C. finmarchicus* stage CV preparing for/in diapause in the regions of GoM, SS, GSL, SLE and NFL. Also shown are results from linear models comparing *PL* among regions and decades within phases. Italic letters (*A*–*D*) below mean values denote significantly different regions (Tukey’s HSD, all *P* < 0.001) within decades, and italic numbers (1–3) and following asterisks denote significantly different decades (Tukey’s HSD, *P* < 0.001 [***], < 0.01 [**], or < 0.05 [*]) within regions. Sample size (N) is shown for each phase in each region, and *F*-test results for the significance of decade and region in each model are shown in last columns and last row, respectively (*P* < 0.001 unless stated otherwise).

Linear models also indicated significant differences in CV copepod *PL* among regions when comparing equivalent phases within decades, with the largest individuals occurring in GSL, and particularly in the estuarine sub-region SLE (Table II, Fig. 2). In the onset phase, individuals in GSL and SLE were larger than in SS in 1990–1999 and larger than in GoM in the two more recent decades (Table II). Similarly, in the diapause phase, individuals in GSL and SLE were larger than in SS in 1990–1999, and in GoM in 2000–2009 (Table II). In general, CV in GoM were smaller than in the other regions, and smaller in the SS than in the GSL regions, where comparable data were available, although the differences between SS and GSL were less pronounced than those between SS and the estuarine subregion (SLE) (Table II). CV in NFL were larger than in most regions in 2000–2009, except for SLE, but in 2010–2020, were unexpectedly smaller than those in GoM, which was the only other region with available data (Table II). The period 2000–2009 had the most extensive dataset that included all regions. In this decade CV decreased in size in the order SLE, NFL, GSL/SS and GoM (Table II).

**Fig. 3:**
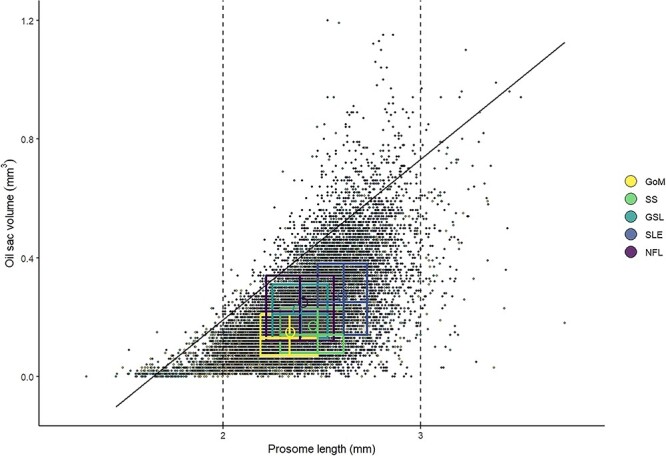
*OSV* (mm^3^) as a function of *C. finmarchicus* stage CV *PL* (mm) in copepods in all sampled areas in the Northwest Atlantic (GoM, SS, GSL, SLE and NFL) from data collected in 2000–2019. Regression line indicates the 0.95 quantile of *OSV*, representing the maximum potential predicted *OSV *(*OSV*_max_) for a given size of copepodite. Dashed lines highlight the copepod size range (2–3 mm) that includes 94% of the observations. Boxes show interquartile range (IQR) and medians for each region and variable, hollow circles show region-specific variable means.

**Fig. 4:**
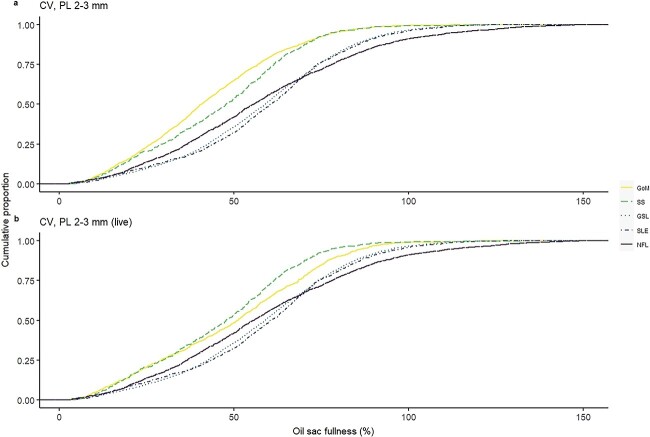
Cumulative distribution of oil sac fullness, expressed as a percentage of the maximum potential *OSV* predicted from quantile regression (%*OSV*_max_), in *C. finmarchicus* stage CV copepodites in lipid-rich phases (onset and diapause) across two decades (2000–2019) in the Northwest Atlantic. Samples are from the selected size class of 2–3 mm including (**a**) all individuals and (**b**) only live-sampled individuals. Regions are GoM, SS, GSL, SLE and NFL.

**Fig. 5:**
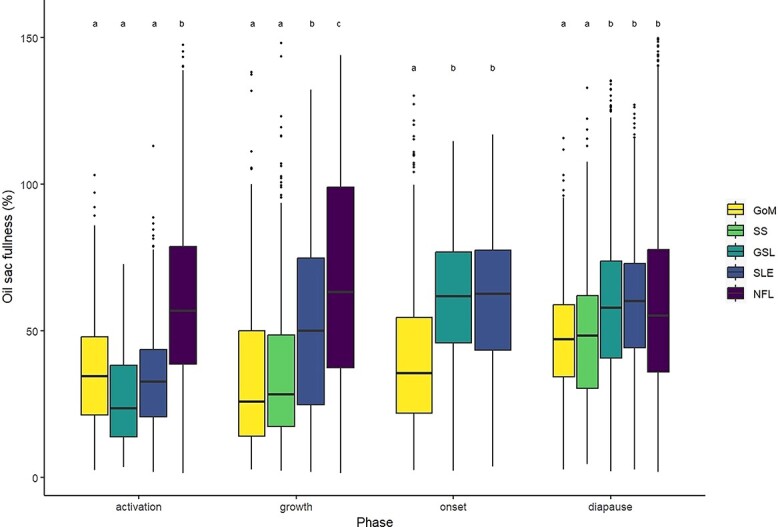
Observations of oil sac fullness, expressed as a percentage of maximum potential OSV predicted from quantile regression (%*OSV*_max_), in *C. finmarchicus* stage CV copepodites in predetermined phases (activation, growth, onset and diapause) over two decades (2000–2019) in the Northwest Atlantic. Upper and lower borders of boxes represent the IQR with median as the horizontal line cutting across and whiskers extending to minimum and maximum values (defined as 1.5*IQR). Points show observations beyond minimum or maximum that were still included in analyses. Formalin-preserved samples and outliers above shown range were excluded. Regions are GoM, SS, GSL, SLE and NFL. Annotated letters (a-c) group together regions within phases that do not have significantly different means (*P* > 0.05).

Quantile regression of *OSV* as a function of *PL* identified a significant effect of *PL* for the 0.95 quantile (*t* = 95.7, *P* < 0.001), with an increase of 0.54 mm^3^ of the 0.95 quantile (maximum potential) of *OSV* for every 1 mm increase in *PL* (Fig. 3). We used the estimated quantile regression equation (*OSV* = β_0_ +β_1*PL*_ + ϵ, where β_0_ = −0.89 and β_1_ = 0.54) to represent maximum oil sac fullness, and found that the proportion of individuals in the onset and diapause phases achieving maximum oil sac fullness (≥100% of *OSV*_max_) was highest in NFL (Fig. 4, Table III), followed by GSL and SLE. Of all regions, GoM and SS had the lowest proportions of the population achieving maximum oil sac fullness in the phases of highest lipid content (0.6 and 0.8%, respectively) (Fig. 4, Table III). The values differed slightly when formalin-preserved samples were excluded, such that higher proportions of CV in GoM, GSL and SLE achieved maximum oil sac fullness (Table III). Up to ~52–64% (SS and GoM, respectively) of the regional populations contained low lipids, while GSL and SLE had the lowest relative amounts of “low lipid” copepods (34.8 and 31.6%, respectively, Table III). Size-adjusted oil sac fullness in live samples in the activation phase was significantly higher in NFL than other regions (*F*_3, 1666_ = 146.6, *P* < 0.001), and similarly in the growth phase, where there was also a significant difference between the more southern regions and SLE (*F*_3, 1724_ = 78.01, *P* < 0.001). The same trend continued in the lipid-rich phases, where oil sac fullness was significantly lower in GoM compared with GSL and SLE in the onset phase (*F*_2, 1041_ = 23.82, *P* < 0.001), and lower in both GoM and SS than other regions in the diapause phase (*F*_4, 8424_ = 29.01, *P* < 0.001) (Fig. 5a). Overall patterns did not change when formalin-preserved samples were included in the analysis (data not shown). Estimated dry weight-specific energy content from lipid [*EC_DW_,* mean (SD)] ranged from a low of 16.05(7.10) J mg^−1^ (GSL) in the activation phase to highs of 27.46(8.22) J mg^−1^ (GSL) in the onset phase and 27.18(10.81) J mg^−1^ (SS) in the diapause phase (Fig. S4).

**Table III:** Maximum potential lipid in copepodites

Region	a) < 50% *OSV*_max_	b) 50–99% *OSV*_max_	c) ≥100% *OSV*_max_
GoM	64.1 (47.7)	35.3 (51.1)	0.6 (1.2)
SS	52	47.2	0.8
GSL	34.8 (35)	61.5 (61.2)	3.7 (3.8)
SLE	31.6 (31.9)	64.2 (63.7)	4.2 (4.4)
NFL	41.3	49.6	9.1

Proportion (%) of sampled *C. finmarchicus* stage CV individuals in the onset or diapause phase in the main size class of *PL* (2–3 mm) accumulating a) low levels (<50%), b) intermediate levels (50–99%) and c) high levels (≥100%) of the maximum potential lipid content (*OSV*_max_) in *OSV* per region (GoM, SS, GSL, SLE and NFL). Values shown in parentheses indicate proportions when formalin-preserved samples were excluded from the analysis. Only live samples were examined from SS and NFL.

Region-specific GLMs indicated temporally consistently higher predicted *OSV* of the average-sized individuals in GSL and SLE compared with the two lower latitude regions (Table IV; Figs S1, S2, onset and diapause phase, respectively). The estimated inferred *TL* content of the oil sac in region-specific average-sized individual copepods ranged from 0.09 mg (GoM, formalin-preserved) to 0.35 mg (SLE) in the onset phase, and 0.12 mg (GoM, formalin-preserved) to 0.34 mg (GSL, live) in the diapause phase (Table IV). Higher *PL* did not always translate to higher inferred lipid in the diapause phase. For example, the average-sized diapause-phase individual in SS (2000–2009) was larger in *PL* than in GoM (2010–2020, live) yet contained lower or equivalent predicted amounts of *TL* (Table IV). Similarly, in decade 1990–1999, the larger diapause phase individuals in SLE contained lower predicted amounts of *TL* compared with GSL individuals in the live sampled population (Table IV). Individual energy content (*EC*_ind_) estimated for an average-sized individual per region ranged from lows of 3.65 J ind^−1^ and 5.40 J ind^−1^ in formalin-preserved and live samples, respectively, in GoM and 5.24 J ind^−1^ in SS, to highs of 13.34 and 13.74 J ind^−1^ in GSL and SLE, respectively (Table IV). The overall regional patterns were similar when *OSV* estimates from 0.5 quantile regressions were used (Table IV, Figs S1–S3).

**Table IV:** Regional lipid and energy metrics in average-sized copepodites

	GLM parameters			*1990–1999*				*2000–2009*				*2010–2020*				*All years*
	**β0**	**β1**	**Null/Residual deviance/d.f.**_**res.dev.**_	*PL* (mm)	*OSV_pred_* (mm^3^)	***TL* (mg)**	***EC***_***ind***_ **(J)**	*PL* (mm)	*OSV_pred_* (mm^3^)	***TL* (mg)**	***EC***_***ind***_ **(J)**	*PL* (mm)	*OSV_pred_* (mm^3^)	***TL* (mg)**	***EC***_***ind***_ **(J)**	***EC***_***ind***_ **(J, mean (SD)**
***Onset***																
GoM (f)	−8.24	2.62	485.14/ 233.43/840					2.36	0.13	**0.11**	**4.49; *4.92***	2.28	0.01	**0.09**	**3.65; *4.11***	**4.07(0.59); *4.52 (0.57)***
GoM (l)	−6.38	1.97	338.68/ 234.09/581					2.36	0.18	**0.16**	**6.32; *7***	2.28	0.15	**0.14**	**5.40; *5.9***	**5.86 (0.65); *6.45 (0.78)***
GSL	−6.81	2.19	98.85/ 56.68/250	2.48	0.25	**0.23**	**8.94; *10.36***	2.63	0.35	**0.31**	**12.41; *13.12***	2.49	0.26	**0.23**	**9.14; *10.54***	**10.16 (1.95); *11.34 (1.54)***
SLE	−6.69	2.10	82.43/ 48.14/260	2.73	0.39	**0.35**	**13.74; *14.57***	2.63	0.31	**0.28**	**11.13; *12.27***					**12.44(1.85); *13.42 (1.62)***
***Diapause***																
GoM (f)	−6.25	1.85	94.11/ 58.25/341					2.27	0.13	**0.12**	**4.62; *4.82***	2.31	0.14	**0.13**	**4.97; *5.19***	**4.80 (0.25); *5.01 (0.26)***
GoM (l)	−6.73	2.16	158.59/ 97.77/385					2.27	0.16	**0.14**	**5.72 *6.33***	2.31	0.18	**0.16**	**6.24; *6.86***	**5.98 (0.37); *6.59 (0.38)***
SS	−6.28	1.91	299.85/ 176.36/627	2.28	0.15	**0.13**	**5.24; *5.91***	2.33	0.16	**0.15**	**5.77; *6.53***					**5.51 (0.37); *6.22 (0.44)***
GSL (f)	−6.18	1.91	9.08/ 6.03/51	2.60	0.30	**0.27**	**10.6; *10.74***	2.35	0.18	**0.17**	**6.58; *5.97***					**8.59 (2.84); *8.36 (3.38)***
GSL (l)	−7.57	2.53	2247.6/ 1210.8/4 356	2.60	0.37	**0.34**	**13.33; *11.53***	2.35	0.20	**0.18**	**7.07; *8.04***					**10.2 (4.43); *9.78 (2.47)***
SLE	−5.58	1.69	352.56/ 251.62/1 006	2.67	0.34	**0.31**	**12.12; *12.68***	2.54	0.27	**0.25**	**9.73; *10.58***					**10.93 (1.69); *11.63 (1.49)***
NFL	−5.74	1.77	1152.8/ 768.5/2165					2.44	0.24	**0.22**	**8.57; *8.75***	2.28	0.18	**0.16**	**6.46; *6.61***	**7.52 (1.49); *7.68 (1.52)***

Model parameters and predicted *OSV* (mm^3^) for an average-sized *C. finmarchicus* stage CV individual in each Northwest Atlantic region (GoM, SS, GSL, SLE and NFL), using region-specific GLM and mean observed *PL* (mm) in the two lipid-rich phases (onset and diapause). Estimated conversion to individual lipid content (mg, *TL*) and individual energy content from lipid (J, *EC*_ind_) are shown in bold adjacent to each predicted *OSV* (*OSV*_pred_). Values in italics indicate *EC*_ind_ estimates derived from regional 0.5 quantile regression models of the *PL*-*OSV* relationship. Separate models were constructed for formalin-preserved (f) and live (l) samples for GoM and for the diapause phase of GSL.

## DISCUSSION

Variations in prey quality among populations can be important for consumers when a single prey species is especially significant or abundant in a particular area and dominates the diet. Habitat choice based on prey abundance and quality can theoretically lead to marked increases in consumer fitness and a selective advantage in terms of survival and growth. Prey quality for zooplanktivorous megafauna, such as NARW, increases with energy richness, which in lipid-rich copepods is characterized by individual size and lipid content. Both maximum potential energy content and realized energy content of *Calanus* spp. copepodites are influenced by environmental history, so that potential energy content, which is driven by body size, is largely controlled by temperature and food concentration (Campbell *et al.*, 2001a), while realized energy content is driven by the seasonal copepod production cycle and food availability. In this study, copepod energy content was evaluated during the lipid-rich phases of the *C. finmarchicus* life cycle, which consisted of stage CV diapause onset and diapause itself. We expected to find increases in copepod body size and inferred lipid content with increasing latitude, mirroring regional variability in sea surface temperature where copepodite development and onset of diapause occur (DFO, 2020), with the caveat that temperature effects could be curtailed by regional differences in food availability.

Using historical data over the span of three decades, we detected patterns in spatial variation, indicating that overall size and energy content of individual CV increased with latitude in the Northwest Atlantic. The CV from the GSL, SLE and NFL were significantly larger in terms of *PL*, and therefore generally had higher size-related inferred lipid and energy contents, than did individuals from lower latitudes in SS and GoM. On a population level, the proportion of CV reaching or exceeding an apparent maximum lipid level was expected to be highest at the onset of diapause and during diapause, because large lipid reserves are needed to meet the metabolic demands of overwintering (Ingvarsdóttir *et al.*, 1999). In SS and GoM regions, considerably fewer of the CV in the average size range of 2–3 mm of sampled individuals were at or over the maximum potential *OSV* during the lipid-rich phases, compared with those in the higher latitude regions of GSL and SLE, and most notably, in the northernmost region of NFL. Along with lower oil sac fullness, this implies that regardless of size, CV were less likely to accumulate lipid up to their maximum potential in the GoM and SS regions, and could be at risk of failure to initiate diapause or of early exit from diapause, especially under warming deep-water conditions (e.g. Maps *et al.*, 2012; Saba *et al.*, 2016), with repercussions for reproduction and survival (Wilson *et al.*, 2016).

We expected *C. finmarchicus* to show regional intra- and interannual variations in size, related to regional differences in the timing of life-cycle events and varying environmental conditions. A long-term trend toward warmer ocean conditions in the Northwest Atlantic regions has been exacerbated by the occurrence of marine heat waves over the past decade, with sea surface temperatures reaching record values across the western north Atlantic continental shelves in summer 2012 (Brickman *et al.*, 2018). Thereafter annual average sea surface temperatures remained higher than normal (the 1981–2010 averages) until 2018, while in 2019, they were near or below normal for the entire area for the first time since 1992 (DFO, 2020). In the GoM and SS, *Calanus* spp. population levels were negatively correlated with rising sea surface temperature (Sorochan *et al.*, 2019), and we anticipated to detect negative effects on body size (Campbell *et al.*, 2001a). However, because of substantial interannual variability in temperature and food conditions and limited observations of copepod size throughout the time series, it was difficult to identify consistent decadal patterns in *PL*. Only GSL and SLE exhibited the hypothesized consistent decrease in *PL* in more recent decades compared with older data, which may imply that mainly temperature, rather than food supply, controlled body size in these regions. In GoM and SS on the other hand, higher *PL* during diapause was observed in more recent decades. To make consistent comparisons among regions, we only compared *PL* within phases, because we expected both lipid-rich phases to include different generations of copepods depending on patterns in regional phenology. For example, individuals in the diapause phase in GoM were likely to consist of both a cohort that begins diapausing early, as well as a later cohort, which would likely be smaller in body size, since it would have developed at higher temperatures and probably lower food concentrations (Durbin *et al.*, 2000). Although the highest *PL* estimates from GSL were based on comparatively small sample sizes, body size in GSL likely largely mirrors that of CV in SLE because of the proximity of the two regions and the transport of active individuals originating from SLE into the GSL (Brennan *et al.*, 2019). It should also be noted, however, that the phase division we used did not account for changes in phenology among decades, such as recent shifts to slightly earlier timing of maximum CV abundance in the GSL (Blais *et al.*, 2021).

Each individual CV has its own trajectory of lipid accumulation, determined by its capacity to store lipid, as well as the quality and quantity of its food. The average-sized individual in the GoM was smaller in *PL* than on the SS, yet lipid content was similar or higher. There were high proportions of individuals at a “low lipid” level in both GoM and SS, but more of the population reached or exceeded *OSV*_max_ in GoM than on the SS. Miller *et al.* (1998) also found that *C. finmarchicus* CV in Georges Bank were often substantially “fatter” in comparison with average individuals in other areas in the North Atlantic. Most of the GoM samples in our study originated from the western GoM and Wilkinson Basin area, which acts as a *C. finmarchicus* depot with some of the highest abundances measured within the species’ range, supplying copepods to the NARW foraging grounds in the Great South Channel and Georges Bank (Melle *et al.*, 2014). The high abundances are a consequence of a transport pathway that regularly deposits offspring of individuals from the Bay of Fundy area to develop through several generations in highly productive and favorable coastal areas of the GoM (Ji *et al.*, 2017), which presumably enables CV lipid accumulation to maximum capacity early in the summer. In our study, lipid content was generally related to body size; however, it was not necessarily proportional to differences in *PL* in southern regions (GoM and SS). Because the lipid storage capacity of copepods is also partly dependent on seasonally varying food conditions (Campbell *et al.*, 2001b), detailed examination of environmental conditions would be needed to determine the influence of regional variation of pre-diapause growth conditions (such as phytoplankton abundance and composition) on the variation in individual CV metrics.

Estimated individual *TL* (0.09–0.35 mg ind^−1^) and *EC* (3.65–13.33 J ind^−1^) values in this study were consistent with previously reported values in the Northwest Atlantic (Miller *et al.*, 1998; Michaud and Taggart, 2007; Davies *et al.*, 2012; McKinstry *et al.*, 2013) and elsewhere (Comita *et al.*, 1966). Although our values are derived from estimated lipid content and not measurements of gross energy content, Davies *et al.* (2012) showed that *OSV*-inferred estimates were comparable with direct measurements for individuals that have substantial lipid stores. Regardless, the estimates made here are most useful as relative comparisons of regional energy content differences. Sorochan *et al.* (2019) suggested that individual *DW* had a limited influence (<20%) on the variation in biomass of late stage *Calanus* spp., with most of the influence stemming from variation in abundance. However, we found that a modest increase in average *PL* (~0.3 mm) can result in a relatively higher increase in estimated energy content, so that individual energy contents in northern regions could be ca. 2–3 times that of those in southern regions. The estimated regional differences in energy content of average-sized individuals at times when NARW are foraging could be substantial enough to influence models that use abundance to estimate required copepod prey densities, as discussed further below.

This study addressed broad patterns of regional variation in *C. finmarchicus* CV size and storage lipid content, but the analysis has limitations arising from use of historical observations from multiple sources, which were not designed to compare regional differences in lipid content. The large dataset had high variability and there were confounding factors, which could not be accounted for in this study. For example, although NFL had the largest proportions of the population reaching *OSV*_max_, there was high intraregional variability with substantial proportions of “low lipid” copepods compared with GSL and SLE. One contributing factor was that the NFL region included diverse sampling points covering a wide latitudinal range (42–55°N) and several subregions (Grand Banks, NFL, Labrador shelf), so that there may have been substantial variability in environmental conditions experienced by individuals in different subregions. In addition, while CV were collected over the entire water column in all study regions, in NFL in particular the population may have included a relatively high proportion of actively feeding individuals from the surface waters, among the diapausing individuals from depth (Pepin and Head, 2009). Indeed, we note that phases were not as clearly defined for the NFL as for the other regions, where patterns in oil sac fullness through the conceptual phases corresponded quite well to what would be expected from phenological data, with both increasing from activation and growth to diapause (Casault *et al.*, 2020b; Blais *et al.*, 2021).

Another source of variability could have been including both live and formalin-preserved samples. Our analysis indicated that separating samples according to preservation state was appropriate for GoM and GSL diapause-phase samples, a result that should be taken into consideration when using the historical dataset for oil sac analyses. Despite the caveats associated with the historical dataset, the large number of observations included in the analysis support accurate characterization of the large-scale patterns of regional differences in size and lipid content, including considerable spatial variation in the relationship between a stable body size metric (*PL*) and inferred lipid content. The resulting intraspecies differences in energetic value could have implications for survival and growth of higher trophic levels. Although the observations in this study did not specifically target NARW foraging areas, our results support the view that regional differences in the capacity for lipid storage by copepods could contribute to the energy content of available prey for NARW.

NARW foraging habitat is characterized by high concentrations of prey at depths shallower than ca. 200 m (Baumgartner *et al.*, 2017; Sorochan *et al.*, 2021). For foraging to be energetically advantageous, the overall prey energy density (prey abundance *x* individual prey energy content) must meet or exceed NARW metabolic requirements, with prey energy density requirements increasing with foraging depth (Gavrilchuk *et al.*, 2020, 2021). NARW have traditionally fed on *C. finmarchicus* and other small copepods in the western GoM from late May to early June, coinciding with the *C. finmarchicus* growth and onset phases in this study. Meanwhile, use of critical foraging habitats in Roseway Basin on the SS in late summer and autumn coincide with the *C. finmarchicus* onset and diapause phases (Kenney *et al.*, 1986). Increasing sightings in the GSL during the late summer and autumn since 2015 (Stokstad, 2017; Simard *et al.*, 2019) are concurrent with the copepod onset phase, and appear to be associated with a shift in foraging distribution co-incident with declines in average abundance of *C. finmarchicus* since 2010 in the GoM and SS (Sorochan *et al.*, 2019).

Potential NARW foraging locations have been correctly predicted from variations in *C. finmarchicus* abundance (Pendleton *et al.*, 2012; Leiter *et al.*, 2017; Plourde *et al.*, 2019) but quantitative estimates of prey energy content are crucial for identifying areas that can energetically sustain whales with varying metabolic costs (Baumgartner and Mate, 2003), allowing females to build fat reserves for reproduction, and to rebuild the endangered population. Prey energy content can influence feeding thresholds that are estimated from bioenergetic models, for example “high lipid” copepods can render a region with moderate copepod abundance suitable for NARW foraging (McKinstry *et al.*, 2013). In essence, prey quality can modulate the effects of prey quantity in a foraging habitat. Thus, although climatological estimates of *C. finmarchicus* abundance in the GoM region have been found to be 2-fold higher than in shallow areas of the GSL (Sorochan *et al.*, 2019), the higher energetic value of the larger copepods can substantially increase the energy yield per foraging effort for NARW in the GSL.

**Fig. 6:**
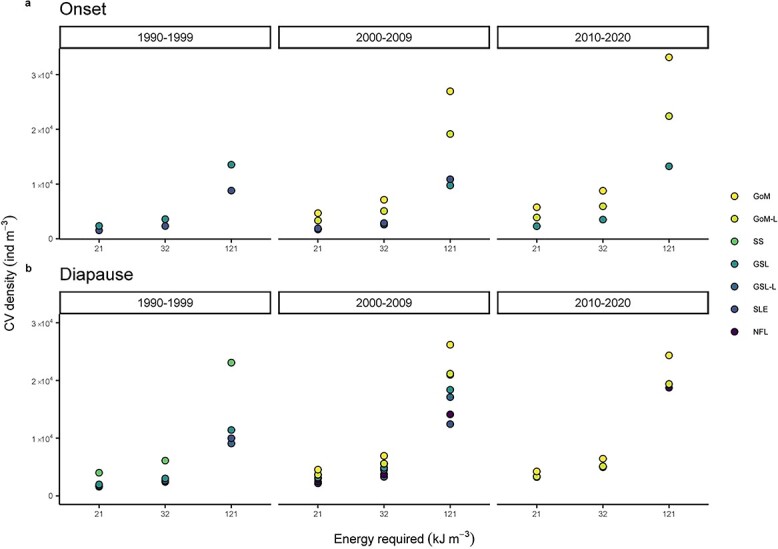
Estimated *C. finmarchicus* CV densities needed to satisfy calculated minimum energy requirements for NARW, ranging from resting individuals in optimal prey conditions (21 kJ m^−3^) to lactating individuals in suboptimal prey conditions (121 kJ m^−3^) (Gavrilchuk *et al.*, 2020, 2021), and an average estimated requirement of 32 kJ m^−3^ (Kenney *et al.*, 1986) in study regions of the northwest Atlantic in 1990–1999, 2000–2009 and 2010–2020 in (**a**) the onset and (**b**) diapause phases. Regions are GoM [GoM-L (live samples only)], SS, GSL [GSL-L (live samples only)], the SLE and NFL.

Minimum NARW energy requirements, and subsequently minimum concentrations of copepods required to energetically sustain NARW, have been estimated in several studies (Kenney *et al.*, 1986; Baumgartner and Mate, 2003; Gavrilchuk *et al.*, 2020, 2021). Without considering inconsistencies in prey depth and density, as our analysis is simplified for constant depth conditions, a theoretical range of ~2 × 10^3^ (SLE) to ~8.8 × 10^3^ (GoM) *C. finmarchicus* CV m^−3^ in the onset phase and a range of ~2.4 × 10^3^ (GSL) to ~6.9 × 10^3^ (GoM) CV m^−3^ in the diapause phase would be required to satisfy a previously estimated minimum energy requirement of ~32 kJ m^−3^ (Kenney *et al.*, 1986) (Fig. 6). More recently, Gavrilchuk *et al.* (2021) estimated minimum prey density thresholds required by NARW in various reproductive states and with varying prey energy content. Using these prey densities, estimated energy requirements ranged from ~21 to 121 kJ m^−3^, depending on reproductive state and the optimality of bioenergetic conditions. The highest estimate would be equivalent to ~33 × 10^3^ CV m^−3^ in GoM compared with ~9 × 10^3^ CV m^−3^ in GSL for lactating NARW (Fig. 6). However, Gavrilchuk *et al.* (2020, 2021) demonstrated that the estimated prey density thresholds would vary with bathymetry due to depth-specific prey density.

Our estimated individual copepod energy contents show up to 3-fold regional differences during the time of highest lipid content and indicate that foraging areas in the GSL are potentially more suitable foraging grounds in late summer compared with previously recognized locations, despite their overall lower copepod densities. In addition, dense aggregations (maximum concentrations of up to 1 × 10^4^ copepods m^−3^) have been observed in late summer in the southern GSL (Sorochan *et al.*, 2023), which has become an important foraging area for NARW since 2010. Such aggregations could provide energy-dense feeding patches (~100 kJ m^−3^), assuming they comprise *C. finmarchicus* CVs in their onset phase, or alternatively *Calanus hyperboreus* stage CIV/V copepodites, which are abundant in the southern GSL (Plourde *et al.*, 2019; Sorochan *et al.*, 2019). The larger *C. hyperboreus* is considered a potentially important NARW prey item in the southern GSL, especially in early summer, and its contribution to overall prey energy should be considered in multispecies *Calanus* regions such as the GSL (Plourde *et al.*, 2001; Lehoux *et al.*, 2020). Although changes in NARW migratory patterns are thought to be driven largely by decreasing biomass of prey in the traditional known foraging areas in GoM and SS (Stokstad, 2017; Sorochan *et al.*, 2019), NARW may be shifting their summer feeding grounds further north to GSL to target the dense aggregations of *Calanus* spp., where higher prey lipid content may further facilitate meeting thresholds for profitable feeding.

## CONCLUSION

The urgency of NARW conservation efforts has prompted research on the factors driving changes in their foraging areas in the NW Atlantic. Uncertainty about NARW distribution during periods of change in their foraging habitat increased mortality from vessel strikes and fishing gear-associated injuries. Regulations have since been put in place to mitigate such interactions in this area, but it would be prudent to try to predict changes in suitable foraging areas. It appears that recent NARW foraging distribution patterns will only be sustained if the feeding conditions are suitable, meaning an abundant supply of late stage (CV) *C. finmarchicus*. Copepod energy content, largely determined by their lipid content, is also important. In this study, we have documented consistent regional differences in *C. finmarchicus* body size and lipid content. Thus, we have surmised that the energetic value of copepods in the newly identified southern GSL foraging area may contribute substantially to its suitability as NARW habitat relative to other foraging habitats where prey quality is lower.

## Supplementary Material

lh_supplemental_materials_v2_fbad047

## Data Availability

The dataset used in this study is available through the Open Government Portal (https://open.canada.ca/data/en/dataset/72e6d3a1-06e7-4f41-acec-e0f1474b555b).
