# Supplementary material for: Spatial variability in size and lipid content of the marine copepod Calanus finmarchicus across the Northwest Atlantic continental shelves: implications for North Atlantic right whale prey quality
Source: J Plankton Res. 2023 Dec 9;46(1):25–40. doi: 10.1093/plankt/fbad047 (PMC10939373; doi:10.1093/plankt/fbad047)
Supplement: lh_supplemental_materials_v2_fbad047 [file lh_supplemental_materials_v2_fbad047.docx]

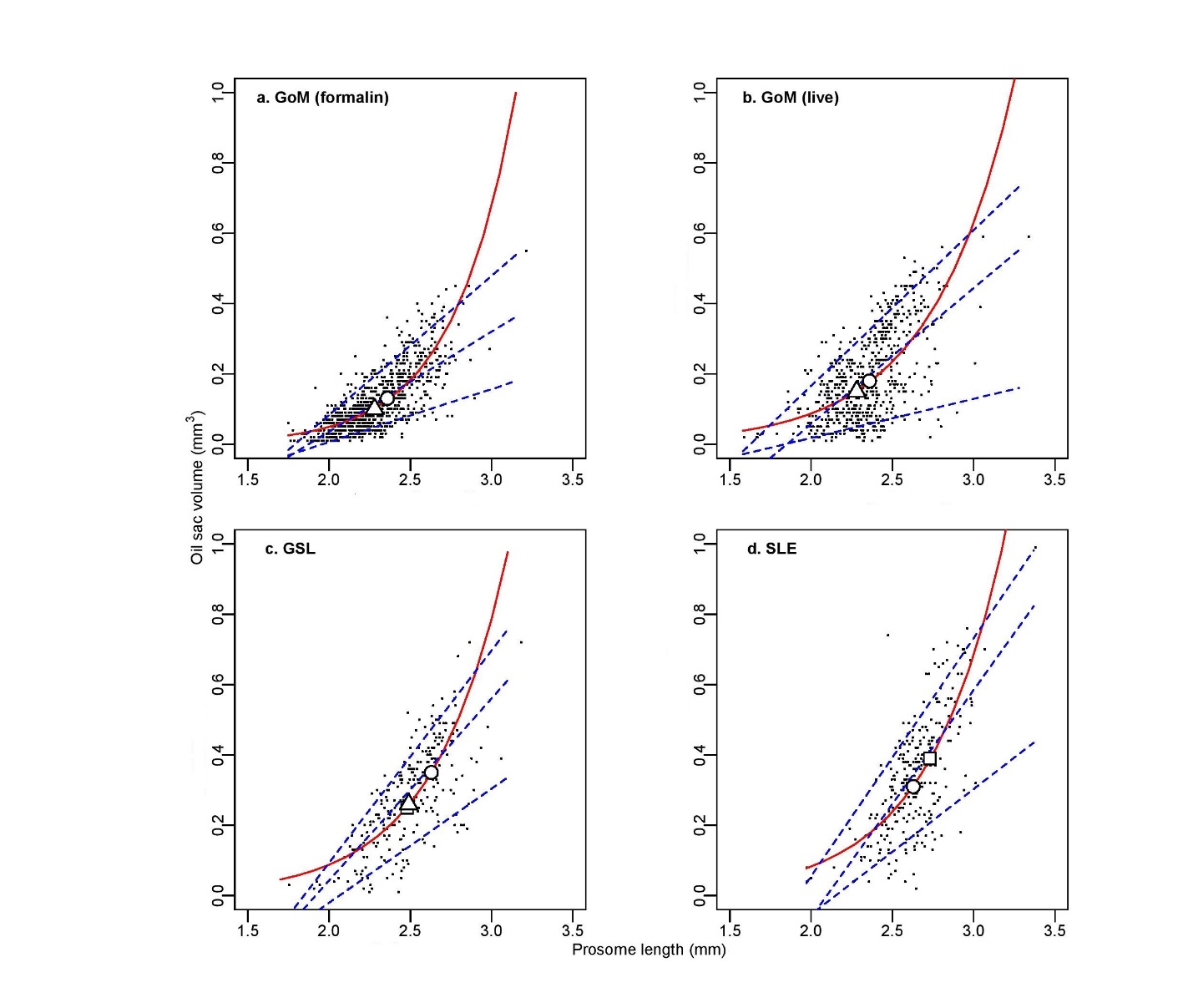


S1. Generalized linear models (red solid lines) and quantile regression models (0.1, 0.5, and 0.9 quantiles; blue dashed lines) of *Calanus finmarchicus* CV oil sac volume (*OSV*, mm^3^) as a function of prosome length (*PL*, mm) in live and formalin-preserved samples in the onset phase in regions of the Northwest Atlantic (Gulf of Maine [GoM, a, b], Gulf of St. Lawrence [GSL, c], and the St. Lawrence Estuary [SLE, d]). Symbols indicate mean *PL* in 1990-1999 (square), 2000-2009 (circle), and 2010-2020 (triangle).


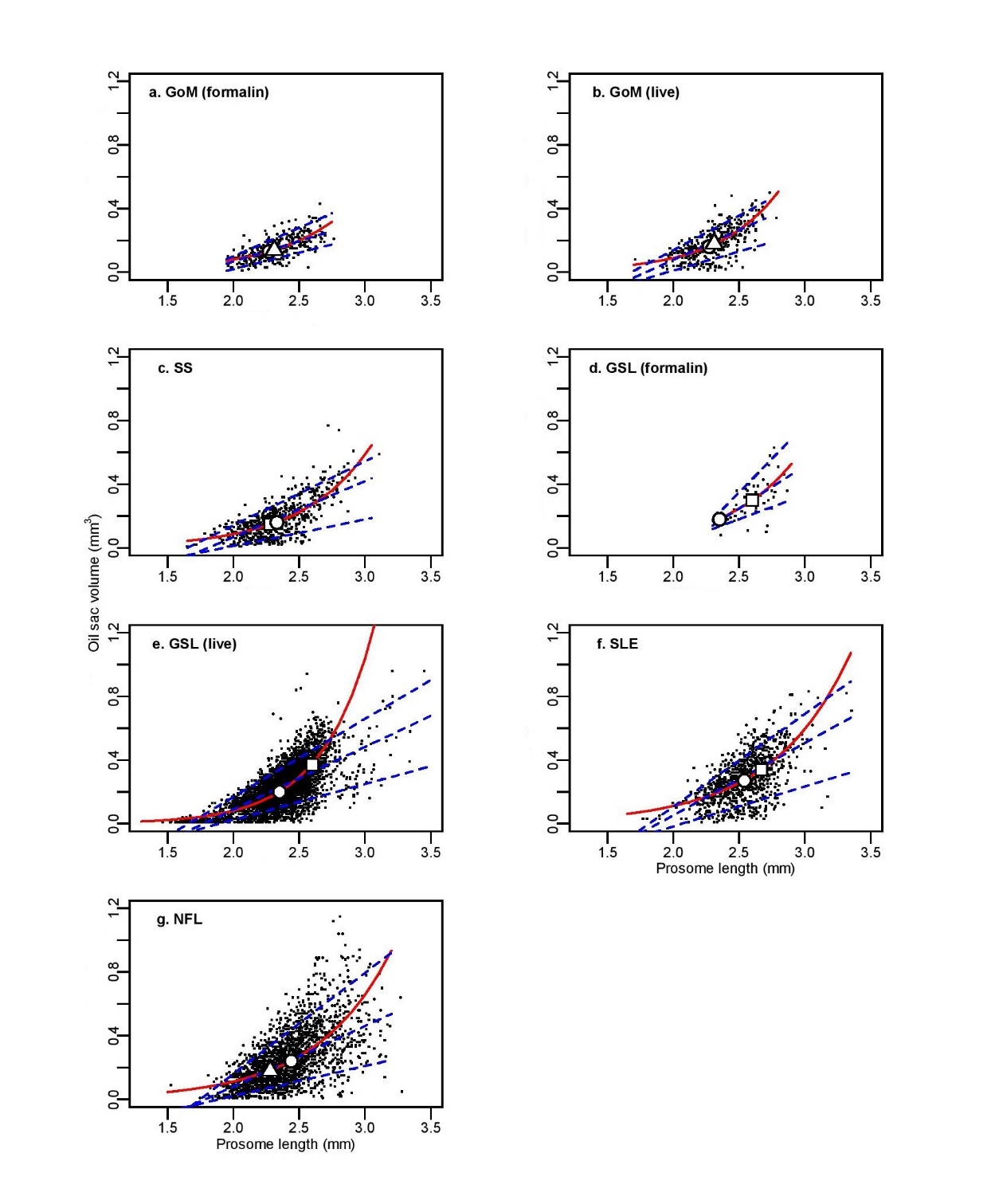


S2. Generalized linear models (red solid lines) and quantile regression models (0.1, 0.5, and 0.9 quantiles; blue dashed lines) of *Calanus finmarchicus* CV oil sac volume (*OSV*, mm^3^) as a function of prosome length (*PL*, mm) in live and formalin-preserved samples in the diapause phase in regions of the Northwest Atlantic (Gulf of Maine [GoM, a, b], Scotian Shelf [SS, c], Gulf of St. Lawrence [GSL, d, e], the St. Lawrence Estuary [SLE, f] and Newfoundland Shelf [NFL, g]). Symbols indicate mean *PL* in 1990-1999 (square), 2000-2009 (circle), and 2010-2020 (triangle).

S3. Predicted oil sac volume (*OSV*, mm^3^) for an average-sized *Calanus finmarchicus* CV in each Northwest Atlantic region (Gulf of Maine [GoM], Scotian Shelf [SS], Gulf of St. Lawrence [GSL], St. Lawrence Estuary [SLE], and Newfoundland Shelf [NFL]). Estimates derived from quantile regression of the 0.1, 0.5 and 0.9 quantiles in the two lipid-rich phases (onset and diapause). Estimated conversion to individual lipid content (mg, *TL*) and individual energy content from lipid (J, *EC_ind_*) are shown adjacent to each predicted *OSV* (*OSV_pred_*). Separate models were constructed for formalin-preserved (f) and live (l) samples for GoM and for the diapause phase of GSL.

| *Onset* | | | | *1990-1999* | | | | *2000-2009* | | | | *2010-2020* | | | | ***All years*** |
| --- | --- | --- | --- | --- | --- | --- | --- | --- | --- | --- | --- | --- | --- | --- | --- | --- |
|  | **Quantile** | **β0** | **β1** | *PL* (mm) | *OSV_pred_* (mm^3^) | ***TL* (mg)** | ***EC_ind_* (J)** | *PL* (mm) | *OSV_pred_* (mm^3^) | ***TL* (mg)** | ***EC_ind_* (J)** | *PL* (mm) | *OSV_pred_* (mm^3^) | ***TL* (mg)** | ***EC_ind_* (J)** | ***EC_ind_* (J,** **mean (SD)** |
| GoM (f) | 0.1 | -0.29 | 0.15 |  |  |  |  | 2.36 | 0.06 | 0.05 | 2.16 | 2.28 | 0.05 | 0.04 | 1.73 | **1.94 (0.30)** |
|  | **0.5** | **-0.53** | **0.28** |  |  |  |  | **2.36** | **0.14** | **0.12** | **4.92** | **2.28** | **0.12** | **0.10** | **4.11** | **4.52 (0.57)** |
|  | 0.9 | -0.71 | 0.40 |  |  |  |  | 2.36 | 0.23 | 0.20 | 8.02 | 2.28 | 0.19 | 0.17 | 6.89 | **7.46 (0.80)** |
| GoM (l) | 0.1 | -0.20 | 0.11 |  |  |  |  | 2.36 | 0.06 | 0.05 | 2.07 | 2.28 | 0.05 | 0.04 | 1.75 | **1.91 (0.22)** |
|  | **0.5** | **-0.71** | **0.39** |  |  |  |  | **2.36** | **0.20** | **0.18** | **7.00** | **2.28** | **0.17** | **0.15** | **5.90** | **6.45 (0.78)** |
|  | 0.9 | -0.72 | 0.44 |  |  |  |  | 2.36 | 0.33 | 0.29 | 11.57 | 2.28 | 0.29 | 0.26 | 10.31 | **10.94 (0.89)** |
| GSL | 0.1 | -0.67 | 0.32 | 2.48 | 0.14 | 0.12 | 4.82 | 2.63 | 0.18 | 0.17 | 6.54 | 2.49 | 0.14 | 0.12 | 4.93 | **5.43 (0.97)** |
|  | **0.5** | **-0.99** | **0.52** | **2.48** | **0.29** | **0.26** | **10.36** | **2.63** | **0.37** | **0.33** | **13.12** | **2.49** | **0.30** | **0.27** | **10.54** | **11.34 (1.54)** |
|  | 0.9 | -1.11 | 0.60 | 2.48 | 0.38 | 0.34 | 13.62 | 2.63 | 0.47 | 0.43 | 16.84 | 2.49 | 0.39 | 0.35 | 13.84 | **14.77 (1.80)** |
| SLE | 0.1 | -0.77 | 0.36 | 2.73 | 0.21 | 0.19 | 7.35 | 2.63 | 0.17 | 0.15 | 6.08 |  |  |  |  | **6.72 (0.90)** |
|  | **0.5** | **-1.36** | **0.65** | **2.73** | **0.41** | **0.37** | **14.57** | **2.63** | **0.35** | **0.31** | **12.27** |  |  |  |  | **13.42 (1.62)** |
|  | 0.9 | -1.30 | 0.68 | 2.73 | 0.55 | 0.49 | 19.52 | 2.63 | 0.48 | 0.43 | 17.12 |  |  |  |  | **18.32 (1.70)** |
| *Diapause* | | | | *1990-1999* | | | | *2000-2009* | | | | *2010-2019* | | | | ***All years*** |
|  | **Quantile** | **β0** | **β1** | *PL* (mm) | *OSV_pred_* (mm^3^) | ***TL* (mg)** | ***EC_ind_* (J)** | *PL* (mm) | *OSV_pred_* (mm^3^) | ***TL* (mg)** | ***EC_ind_* (J)** | *PL* (mm) | *OSV_pred_* (mm^3^) | ***TL* (mg)** | ***EC_ind_* (J)** | ***EC_ind_* J,** **mean (SD)** |
| GoM (f) | 0.1 | -0.40 | 0.21 |  |  |  |  | 2.27 | 0.08 | 0.07 | 2.67 | 2.31 | 0.08 | 0.08 | 2.97 | **2.82 (0.21)** |
|  | **0.5** | **-0.44** | **0.26** |  |  |  |  | **2.27** | **0.14** | **0.12** | **4.82** | **2.31** | **0.15** | **0.13** | **5.19** | **5.01 (0.26)** |
|  | 0.9 | -0.62 | 0.36 |  |  |  |  | 2.27 | 0.20 | 0.18 | 7.00 | 2.31 | 0.21 | 0.19 | 7.51 | **7.25 (0.36)** |
| GoM (l) | 0.1 | -0.47 | 0.24 |  |  |  |  | 2.27 | 0.07 | 0.07 | 2.64 | 2.31 | 0.08 | 0.08 | 2.98 | **2.81 (0.24)** |
|  | **0.5** | **-0.67** | **0.37** |  |  |  |  | **2.27** | **0.18** | **0.16** | **6.33** | **2.31** | **0.19** | **0.17** | **6.86** | **6.59 (0.38)** |
|  | 0.9 | -0.73 | 0.43 |  |  |  |  | 2.27 | 0.26 | 0.23 | 9.10 | 2.31 | 0.27 | 0.25 | 9.72 | **9.41 (0.44)** |
| SS | 0.1 | -0.32 | 0.17 | 2.28 | 0.06 | 0.05 | 2.07 | 2.33 | 0.07 | 0.06 | 2.37 |  |  |  |  | **2.22 (0.21)** |
|  | **0.5** | **-0.64** | **0.35** | **2.28** | **0.17** | **0.15** | **5.91** | **2.33** | **0.18** | **0.17** | **6.53** |  |  |  |  | **6.22 (0.44)** |
|  | 0.9 | -0.67 | 0.40 | 2.28 | 0.25 | 0.23 | 9.01 | 2.33 | 0.27 | 0.25 | 9.73 |  |  |  |  | **9.37 (0.51)** |
| GSL (f) | 0.1 | -0.58 | 0.30 | 2.6 | 0.21 | 0.19 | 7.50 | 2.35 | 0.14 | 0.12 | 4.80 |  |  |  |  | **6.15 (1.91)** |
|  | **0.5** | **-1.09** | **0.54** | **2.6** | **0.30** | **0.27** | **10.74** | **2.35** | **0.17** | **0.15** | **5.97** |  |  |  |  | **8.36 (3.38)** |
|  | 0.9 | -1.76 | 0.84 | 2.6 | 0.43 | 0.39 | 15.42 | 2.35 | 0.22 | 0.20 | 7.92 |  |  |  |  | **11.67 (5.31)** |
| GSL (l) | 0.1 | -0.42 | 0.22 | 2.6 | 0.16 | 0.14 | 5.64 | 2.35 | 0.10 | 0.09 | 3.65 |  |  |  |  | **4.64 (1.41)** |
|  | **0.5** | **-0.70** | **0.39** | **2.6** | **0.32** | **0.29** | **11.53** | **2.35** | **0.23** | **0.20** | **8.04** |  |  |  |  | **9.78 (2.47)** |
|  | 0.9 | -0.81 | 0.49 | 2.6 | 0.46 | 0.42 | 16.41 | 2.35 | 0.34 | 0.31 | 12.05 |  |  |  |  | **14.23 (3.08)** |
| SLE | 0.1 | -0.52 | 0.25 | 2.67 | 0.15 | 0.14 | 5.36 | 2.54 | 0.12 | 0.11 | 4.20 |  |  |  |  | **4.78 (0.82)** |
|  | **0.5** | **-0.86** | **0.46** | **2.67** | **0.36** | **0.32** | **12.68** | **2.54** | **0.30** | **0.27** | **10.58** |  |  |  |  | **11.63 (1.49)** |
|  | 0.9 | -1.06 | 0.58 | 2.67 | 0.50 | 0.45 | 17.67 | 2.54 | 0.42 | 0.38 | 14.97 |  |  |  |  | **16.32 (1.91)** |
| NFL | 0.1 | -0.36 | 0.19 |  |  |  |  | 2.44 | 0.10 | 0.09 | 3.65 | 2.28 | 0.07 | 0.07 | 2.58 | **3.12 (0.76)** |
|  | **0.5** | **-0.68** | **0.38** |  |  |  |  | **2.44** | **0.25** | **0.22** | **8.75** | **2.28** | **0.19** | **0.17** | **6.61** | **7.68 (1.52)** |
|  | 0.9 | -1.11 | 0.63 |  |  |  |  | 2.44 | 0.44 | 0.39 | 15.49 | 2.28 | 0.33 | 0.30 | 11.90 | **13.69 (2.54)** |


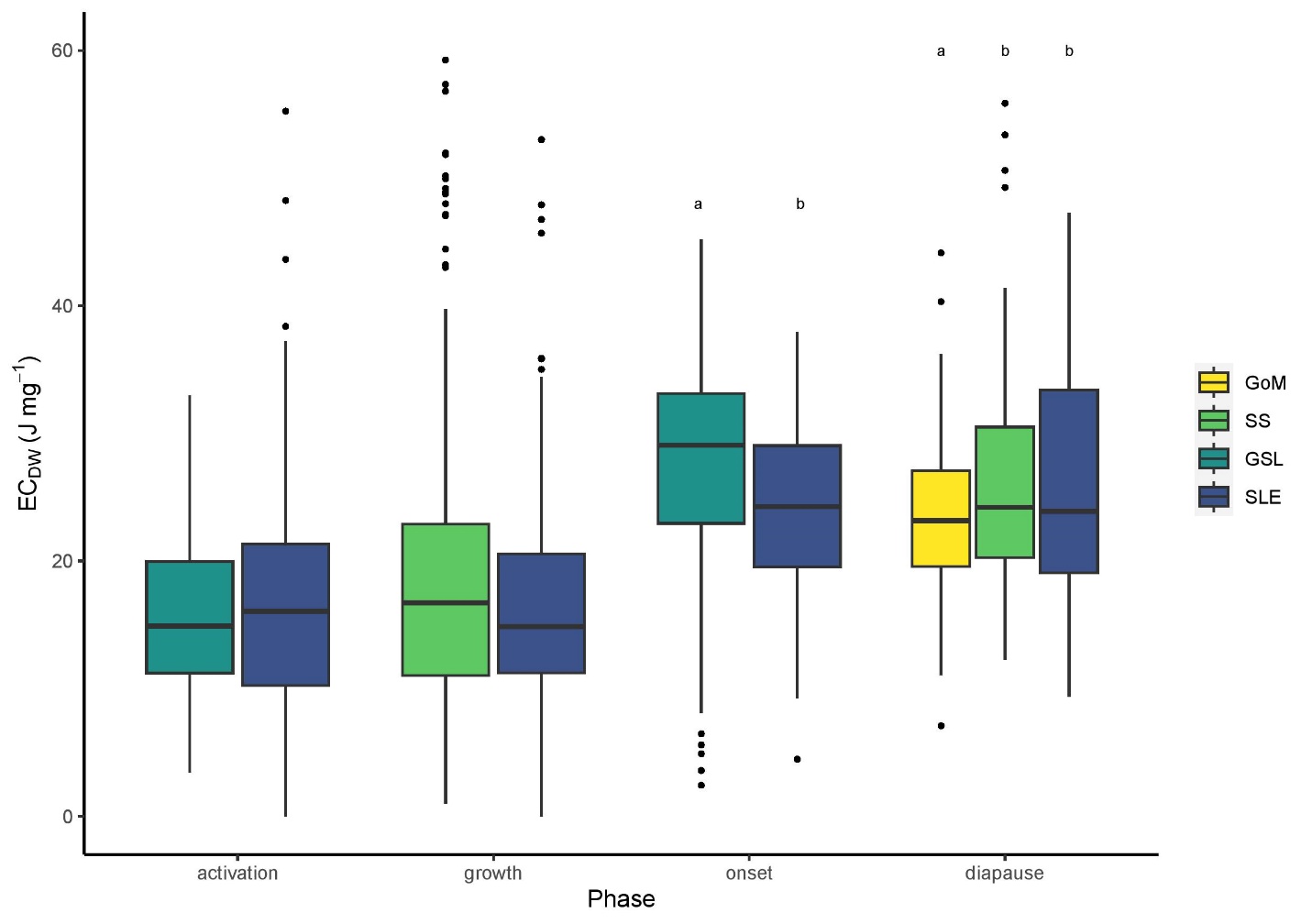


S4. Dry weight-specific energy content from lipid (*EC_DW_*, J mg^-1^) in *Calanus finmarchicus* CV copepodites in predetermined phases (activation, growth, onset, diapause) over two decades (2000-2019) in the Northwest Atlantic. Upper and lower borders of boxes represent the interquartile range (IQR) with median as the horizontal line cutting across and whiskers extending to minimum and maximum values (defined as 1.5*IQR). Points show observations beyond minimum and maximum that were still included in analyses. Formalin-preserved samples and outliers above shown range were excluded. Regions are Gulf of Maine (GoM), Scotian Shelf (SS), Gulf of St. Lawrence (GSL), and St. Lawrence Estuary (SLE). Annotated letters (a-b) group together regions within phases that did not have significantly different means (*p* > 0.05) when overall differences were found.
